# Pupil-light reflex in early-stage Parkinson's disease

**DOI:** 10.1177/1877718X261436290

**Published:** 2026-04-08

**Authors:** Solveig EJ Dalbro, Lasse Pihlstrøm, Emilia Kerty, Mathias Toft

**Affiliations:** 1 Department of Neurology, Oslo University Hospital, Oslo, Norway; 2 Institute of Clinical Medicine, University of Oslo, Oslo, Norway

**Keywords:** Parkinson, pupil, orthostatic, hypotension, sympathetic nervous system, parasympathetic nervous system, eye tracker

## Abstract

We examined pupil-light reflex in 25 drug-naïve patients with newly diagnosed Parkinson's disease and compared them to 29 healthy controls. We also evaluated effects of symptomatic treatment and correlations with clinical scores. Besides a smaller pupil diameter at maximum constriction, no pupil parameter robustly distinguished patients from controls. Following initiation of symptomatic therapy, baseline pupil diameter and constriction amplitude increased, whereas peak constriction velocity remained unchanged and correlated with patient-reported autonomic symptoms. These findings suggest peak constriction velocity may reflect autonomic dysfunction less influenced by medication. Further studies should clarify the pupil-light reflex's clinical utility in Parkinson's disease.

**Plain Language Summary Title:**

Pupil response to light in newly diagnosed Parkinson's patients

## Plain Language Summary

We tested pupil responses to light in 25 newly diagnosed, unmedicated Parkinson's patients and 29 healthy people, then retested patients after starting Parkinson's treatment. Pupil responses did not clearly detect early Parkinson's. After treatment, resting pupil size increased, but peak constriction velocity did not change and was linked to patients' autonomic symptoms. Peak constriction velocity may be a medication-resistant marker of autonomic dysfunction and deserves study in larger trials.

## Introduction

Biomarkers are needed in Parkinson's disease to support earlier diagnosis and personalized disease monitoring and therapy. Autonomic dysfunction is a common non-motor symptom in Parkinson's disease and may precede clinical diagnosis.^1^ Measurement of the pupil-light reflex provides a non-invasive method for studying autonomic function. Previous studies report delayed and reduced constriction velocity and amplitude in established Parkinson's disease versus healthy controls,^2–5^ with changes across disease stages.^6^ We examined pupil responses in drug-naïve patients and controls, then after initiation of symptomatic treatment and after one year, to determine whether pupil alterations occur early and respond to treatment.

## Materials and methods

Participants were from the Prospective Study of Parkinsonism in Oslo (PROSPOS), with regional ethics approval and written informed consent. We compared drug-naïve Parkinson's disease patients (n = 25; mean age 67.9 years; eleven females) meeting the Movement Disorder Society clinical diagnostic criteria for probable or established Parkinson's disease^7^ to age-matched healthy controls (n = 29, mean age 64.1 years; 20 females). Participants with glaucoma or diabetes were excluded from analyses. Clinical data included Movement Disorder Society-Unified Parkinson's Disease Rating Scale **(**MDS-UPDRS), Sniffin’ Sticks 12-item smell identification test, the Montreal Cognitive Assessment (MoCA), supine and 3-min standing blood pressure, and Scales for Outcomes in Parkinson's Disease – Autonomic questionnaire (SCOPA-AUT).^8^ Patients who consented were re-evaluated after approximately 3 and 12 months with MDS diagnostic criteria, medication status and repeated eye movement recordings.

Pupil dynamics were measured with BulbiCAM (BulbiTech, Trondheim, Norway) a 400 frames per second eye-tracking device.^9^ Participants fixated a central red target; both monitors displayed 5 candela per square meter (cd/m^2^) dark screens for 3 s, 300 cd/ m^2^ bright screens for 2 s, then 5 cd/m^2^ dark for 3 s. Files from BulbiCAM were analysed in R version 4.1.2.^10^ Metrics analysed were baseline diameter, prestimulus diameter, max constriction diameter, constriction amplitude (prestimulus diameter – max constriction diameter in absolute diameter), latency in ms and constriction peak velocity in mm per second.

Group comparisons used Welch's t-test for normally distributed variables and Wilcoxon signed-rank test for non-normal variables. Paired pre/post treatment and baseline versus 12 months differences were analysed with paired t-tests or Wilcoxon signed-rank test. Significance level was 0.05. No correction for multiple comparisons of pupil variables was applied, given their interrelation and a focused hypothesis-driven analysis.^2–5^ Correlations with orthostatic blood pressure, MDS-UPDRS and SCOPA-AUT used Pearson's r for normal and Spearman's ρ for non-normal variables; likewise, these were also hypothesis-driven and unadjusted.^2,4^

## Results

The patients were newly diagnosed with a mean of 42 days since diagnosis, and the mean MDS-UPDRS motor score was 17. Orthostatic hypotension was found on examination in three patients, and mean SCOPA-AUT score was 12. Max constriction diameter was significantly smaller in patients compared to controls. No significant differences between groups were found for the remaining pupil diameters, constriction latency, or peak velocity (Table 1). Higher SCOPA-AUT scores correlated with slower peak velocity (r = 0.54, *p* = 0.005, CI 0.18–0.77). By the second visit, seventeen patients had initiated symptomatic, mainly dopaminergic, treatment. Treatment regimens included two patients on pramipexole with selegiline, one on rasagiline monotherapy, one on a combination of rasagiline and levodopa, eleven on levodopa monotherapy and the remaining two on levodopa in combination with selegiline. The mean levodopa equivalent daily dose (LEDD) was 247 mg at 3 months and 344 mg at 12 months. In exploring the effect of symptomatic therapy on pupil variables, we found that baseline diameter and constriction amplitude were increased after initiation of treatment (3 months) (Table 1). After one year the mydriatic effect on the baseline diameter persisted while the other variables were stable (Table 1).

## Discussion

We found a significant difference in max constriction diameter, but no differences between patients and controls in the remaining pupil-light reflex parameters. Previous studies, mostly in well-established patients on treatment, reported increased pupil constriction latency,^3,4^ decreased constriction amplitude,^3,4,11^ and decreased peak velocity.^2,4,11^ Although these studies do not fully replicate one another, there is a trend toward increased latency and decreased constriction amplitude and peak velocity. In our study the trends align but do not reach statistical significance, possibly due to limited statistical power. These findings suggest that alterations in the pupil-light reflex may be subtle and contingent upon test conditions in early disease. After initiation of symptomatic treatment, baseline pupil diameter and constriction amplitude increased, replicating a mydriatic effect reported previously both in mice,^12^ healthy individuals^13^ and in Parkinson's disease patients on levodopa/carbidopa.^14,15^ By contrast, peak velocity correlated with autonomic symptom burden (SCOPA-AUT) and remained unchanged after initiation of treatment and at 12 months. Strengths of our study include assessment in drug-naïve patients and re-evaluation after treatment and after one year; limitations include the small sample size and the low dose mixed treatment regimens. In conclusion, peak velocity shows potential as a treatment-insensitive marker of autonomic, mainly parasympathetic, dysfunction in Parkinson's disease. Larger, longitudinal studies are needed to confirm clinical utility of the pupil-light reflex in Parkinson's disease.

**Table 1.** Pupil diameter, latency and peak velocity in Parkinson's disease (PD) patients and healthy controls at baseline, and in the treated PD subgroup at baseline, 3 months, and 12 months.

Variable (unit)	Patients baseline (n = 25)	Controls baseline (n = 29)	Before treatment (n = 17)	After treatment 3 months (n = 17)	After treatment 12 months (n = 17)
Baseline diameter (mm)	3.01 ± 0.7	3.31 ± 0.6	2.95 ± 0.7	3.12 ± 0.7^a^	3.20 ± 0.76^b^
Prestimulus diameter (mm)	3.46 ± 0.8	3.78 ± 0.6	3.51 ± 0.8	3.61 ± 0.8	3.55 ± 0.79
Max constriction diameter (mm)	2.13 ± 0.5	2.31 ± 0.3^c^	2.09 ± 0.4	2.12 ± 0.4	2.12 ± 0.37
Constriction amplitude (mm)	1.34 ± 0.4	1.48 ± 0.4	1.42 ± 0.5	1.48 ± 0.5^d^	1.43 ± 0.57
Latency (ms)	317 ± 32	305 ± 24	322 ± 35	319 ± 23	324 ± 64
Peak Velocity (mm/s)	−3.95 ± 1	−4.16 ± 0.7	−4.11 ± 1.0	−4.08 ± 1.1	−4.42 ± 1.27

Values are mean ± standard deviation. Mm, millimetres; ms, milliseconds; mm/s, millimetres per second.

^a^ Baseline vs 3 months, *p* = 0.003 (paired t-test)

^b^ Baseline versus 12 months *p* = 0.01 (Wilcoxon signed-rank test)

^c^ Patients versus controls *p* = 0.047 (Wilcoxon signed-rank test)

^d^ Baseline versus 3 months *p* = 0.04 (paired t-test)
